# FS-FCN: a frequency–spatial fusion convolutional network for brain tumor classification in MR images

**DOI:** 10.3389/fneur.2026.1948996

**Published:** 2026-09-08

**Authors:** Kun Zhang, Menghao Zhao, Jincan Zhang, Wenna Chen, Ganqin Du

**Affiliations:** 1 The First Affiliated Hospital, and College of Clinical Medicine of Henan University of Science and Technology, Luoyang, China; 2 College of Information Engineering, Henan University of Science and Technology, Luoyang, China

**Keywords:** brain tumor classification, convolutional neural networks, frequency–spatial fusion, magnetic resonance imaging, wavelet transform

## Abstract

**Introduction:**

Brain tumor classification using magnetic resonance imaging (MRI) is important for computer-aided medical image analysis. However, different brain tumor types may exhibit highly similar grayscale patterns, textures, and boundaries. Conventional convolutional neural networks rely primarily on spatial-domain convolution and do not explicitly model frequency-domain information. Moreover, high-frequency details, such as edges and textures, are often weakened by multi-stage downsampling, while interaction between spatial- and frequency-domain features remains limited. Consequently, fine-grained differences among tumor categories may not be effectively captured.

**Methods:**

To address these limitations, a Frequency–Spatial Fusion Convolutional Network (FS-FCN) was developed for brain tumor MRI classification. A Parametric Wavelet Downsampling (PWD) module and a Frequency–Spatial Convolution (FSConv) module were incorporated into the convolutional network. Spatial- and frequency-domain information was jointly modeled during multilevel encoding and deep feature learning to improve the preservation and discriminative representation of key structures, local textures, and edge details.

**Results:**

On the Cheng Brain Tumor MRI Three-Class Classification Dataset, FS-FCN achieved an average classification accuracy of 98.53% ± 0.35% using five-fold cross-validation. The applicability of the model across different classification settings was further evaluated through a four-class classification experiment.

**Discussion:**

The results demonstrate that jointly modeling spatial- and frequency-domain information improves the representation of brain tumor MRI features and provides an effective feature-learning approach for brain tumor MRI classification.

## 1 Introduction

Brain tumors are neurological disorders that pose a serious threat to human health. Abnormalities in the structure and function of brain tissue may be caused by these tumors, substantially affecting patients’ health (1). Based on their origin, brain tumors are generally classified as primary or secondary. Primary tumors arise from intracranial tissues, including the brain, meninges, and pituitary gland, whereas secondary tumors result from the metastasis of malignant tumors originating elsewhere in the body (2). According to tissue origin and pathological type, common brain tumors include gliomas, meningiomas, and pituitary tumors. These tumor types differ in anatomical location, tissue origin, and magnetic resonance imaging (MRI) characteristics. However, similar textures, morphologies, and boundary patterns may be observed across certain tumor types, making accurate classification challenging (3, 4). Therefore, efficient and accurate methods for brain tumor image classification are needed to support automated medical image analysis and advance intelligent image-processing technologies.

MRI has become one of the primary imaging techniques used for brain tumor detection and classification because of its excellent soft-tissue contrast and detailed anatomical information (5). Multidimensional information about tumor location, morphology, and internal structure can be provided by MRI, offering an important basis for lesion assessment and tumor classification (6). However, conventional MRI diagnosis relies heavily on the expertise of radiologists and is therefore time-consuming and susceptible to subjective interpretation. In particular, different tumor types, including gliomas, meningiomas, and pituitary tumors, may exhibit similar grayscale distributions, textures, and structural patterns. Consequently, discriminative imaging features may not be fully identified through manual examination alone (7). Therefore, the development of automated and intelligent methods for brain tumor MRI classification has become an important research direction in medical image analysis.

In recent years, Convolutional Neural Networks (CNNs) have been widely applied to medical image analysis owing to advances in deep learning (8). Unlike traditional methods that rely on handcrafted features, CNNs can automatically learn hierarchical representations through multiple nonlinear transformations, enabling the effective extraction of edges, textures, and high-level semantic features (9). Transformers also offer advantages in modeling global dependencies through self-attention. However, their local inductive bias is relatively limited, and their performance often depends heavily on large-scale datasets and pretraining (10). Therefore, CNN-based methods remain a viable approach to brain tumor MRI classification. Nevertheless, conventional CNNs primarily extract features through spatial convolution and pooling, with limited explicit modeling of frequency-domain information (11). Moreover, image details and structural information may be progressively lost as the network deepens and multi-stage downsampling is performed. Consequently, tumor boundaries, local textures, and other fine-grained features may not be adequately preserved (12). These limitations reduce the model’s ability to distinguish subtle imaging differences among brain tumor types.

To address these limitations, attention mechanisms, multiscale feature fusion, and frequency-domain analysis have been increasingly explored to improve feature representation. Attention mechanisms allow the importance of different spatial regions or feature channels to be adaptively weighted. Multiscale structures enable semantic information at different scales to be integrated, while frequency-domain analysis characterizes image structures across different frequency components (13, 14). However, existing methods mainly focus on improving feature extraction, whereas the information loss caused by downsampling during network encoding has received limited attention (15). Moreover, spatial- and frequency-domain features are often modeled independently, resulting in insufficient information exchange between the two domains. Therefore, preserving key structural information during feature downsampling and effectively integrating local details with global structural information remain critical challenges in brain tumor MRI classification.

To address these issues, a Frequency–Spatial Fusion Convolutional Network (FS-FCN) is proposed for brain tumor MRI image classification. A Parametric Wavelet Downsampling (PWD) module and a Frequency–Spatial Convolution (FSConv) module are incorporated to jointly model spatial- and frequency-domain information during multilevel encoding and deep feature learning. Key details are preserved during progressive downsampling, while local texture and global structural differences are more effectively represented.

The main contributions of this paper are as follows:

- A Frequency–Spatial Fusion Convolutional Network (FS-FCN) was developed to unify frequency-domain information preservation during downsampling with deep spatial–frequency feature fusion.
- The PWD module was incorporated into the network to preserve key structural and textural information during feature downsampling by integrating wavelet subband information with spatial convolutional features.
- The FSConv module was applied during deep feature extraction to improve the representation of fine-grained differences among brain tumor categories through high-frequency feature extraction, low-frequency structural modeling, and spatial–frequency feature interaction.
- The effectiveness of the model was comprehensively evaluated on a three-class dataset using multiple evaluation metrics and experimental analyses, and its applicability across different classification settings was further validated on a four-class dataset.

## 2 Related work

Early methods for brain tumor MRI classification relied primarily on traditional machine learning algorithms. Image information was typically represented using handcrafted features, which were then combined with classifiers to predict tumor categories. Because MRI images contain rich textural and structural information, grayscale, texture, and statistical features were commonly extracted for image representation. For example, Usman and Rajpoot (16) constructed an MRI feature representation based on intensity, local neighborhood, and wavelet texture features, which was combined with a random forest classifier to classify brain tumor regions. Chinnam et al. (17) integrated texture and Gabor wavelet features and employed a PUK-SVM classifier for brain tumor identification. However, the performance of these methods depends heavily on handcrafted feature design and domain-specific prior knowledge, limiting their ability to capture high-level semantic information from complex MRI images.

With advances in deep learning, CNNs have become a major approach to brain tumor MRI classification. Unlike traditional machine learning methods, CNNs can automatically learn hierarchical feature representations through multilayer convolutional structures, reducing dependence on handcrafted features and enabling richer spatial information to be extracted from MRI images. For example, Badža et al. (18) developed a CNN-based method in which features were automatically extracted from MRI images to classify gliomas, meningiomas, and pituitary tumors. Sultan et al. (19) further developed a deep CNN with increased network depth to improve feature learning and the discrimination of different brain tumor types. Kabir et al. (20) integrated a CNN with a genetic algorithm to optimize the network architecture for MRI-based glioma grading and classification, further demonstrating the effectiveness of deep learning in brain tumor image analysis. However, conventional CNNs rely primarily on spatial-domain convolution and do not explicitly model frequency-domain information. Moreover, edge, texture, and other fine-grained structural information may be progressively weakened by successive convolution and downsampling operations, thereby limiting the discrimination of visually similar tumor types.

To further improve the feature selection capability of CNNs, attention mechanisms have been increasingly incorporated into convolutional networks. Unlike conventional convolutional operations, in which all features are processed uniformly, attention mechanisms allow feature weights to be adaptively adjusted according to their importance, thereby improving the use of discriminative information. For example, Hu et al. (21) proposed the Squeeze-and-Excitation (SE) module, in which channel-wise features are recalibrated by explicitly modeling interchannel dependencies, thereby enhancing feature representation. Wang et al. (22) subsequently proposed the Efficient Channel Attention Network (ECA-Net), in which channel modeling is improved through local cross-channel interactions while low computational complexity is maintained. These channel attention methods provide effective mechanisms for feature selection in convolutional networks. However, they primarily reweight existing features and cannot directly recover detailed information lost during convolution and downsampling.

In recent years, frequency-domain information has increasingly been incorporated into deep learning networks to improve the representation of image textures, edges, and multiscale structures. Unlike spatial-domain features, frequency-domain information characterizes image variations across different frequency components. Low-frequency components mainly preserve global structural information, whereas high-frequency components contain rich edge and texture details. For example, Amin et al. (23) applied the Discrete Wavelet Transform (DWT) to fuse multimodal MRI sequences and combined the resulting features with a CNN for brain tumor classification. By integrating information from different frequency subbands, the representation of structural and textural features was enhanced. Zhao et al. (24) further incorporated wavelet transforms into medical image analysis networks. Images were decomposed into low- and high-frequency subbands to reduce the loss of frequency information during conventional downsampling, while an attention mechanism was used to enhance feature representation.

Based on the studies reviewed above, traditional machine learning methods rely heavily on handcrafted features. Although CNNs and attention mechanisms have improved feature learning, frequency-domain information remains underutilized. Therefore, frequency-domain analysis has been increasingly incorporated into deep learning networks to enhance the representation of image structures and textures. However, preserving key frequency information during multilevel encoding and enabling effective interactions between spatial- and frequency-domain features remain insufficiently explored. To address these limitations, an FS-FCN is proposed. Spatial- and frequency-domain information is jointly modeled throughout multilevel encoding and deep feature learning. Consequently, global structures, local textures, and edge details can be effectively represented as the spatial resolution of the feature maps is progressively reduced, establishing a unified frequency–spatial feature-learning framework across the encoding process.

## 3 Proposed method

### 3.1 Overall structure of FS-FCN

**Figure 1:**
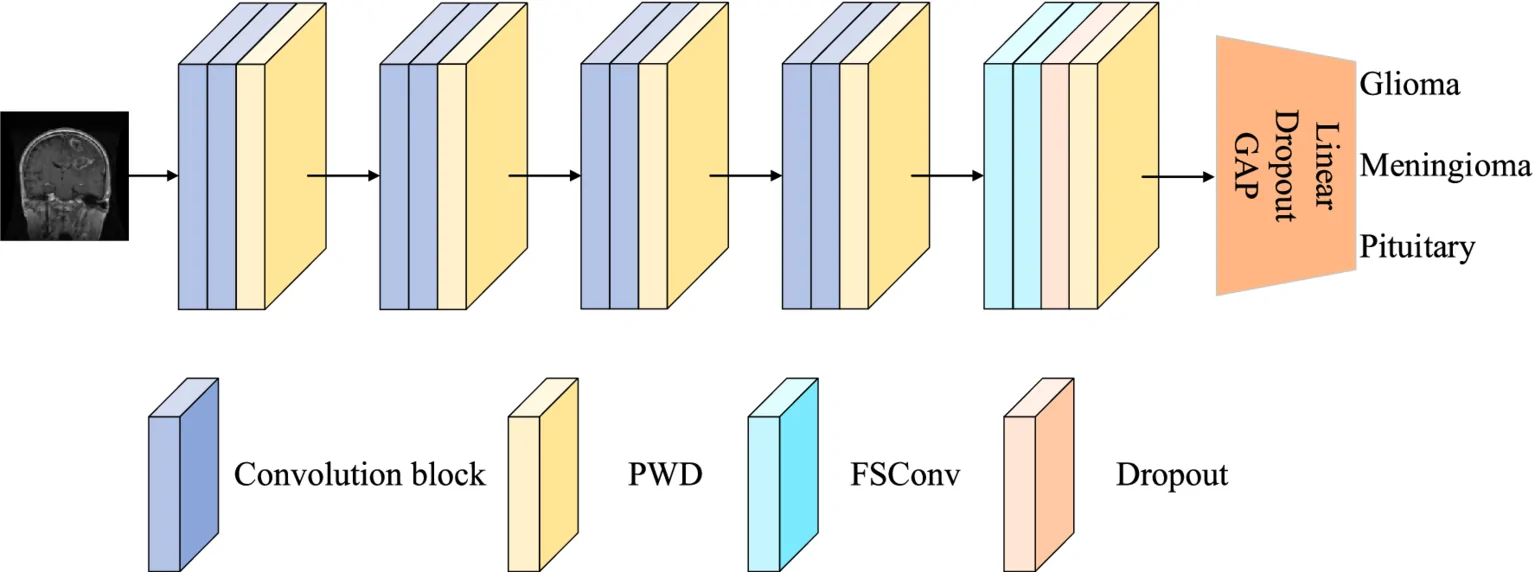
Overall architecture of the FS-FCN network.

An FS-FCN is proposed for brain tumor MRI image classification. As shown in Figure 1, the network mainly consists of convolutional blocks, PWD modules (25), FSConv modules (26), and a classifier.

The input MRI images are first processed by convolutional blocks to encode shallow spatial features. The spatial resolution is then progressively reduced, while the number of channels is increased through four encoding stages, each composed of convolutional blocks and PWD modules. Compared with conventional max-pooling and strided convolution, PWD jointly models wavelet- and spatial-domain information, allowing structural and textural features to be better preserved during downsampling. After multilevel encoding, two cascaded FSConv modules are applied to the deep features for wavelet decomposition and spatial–frequency feature interaction. This process improves the joint representation of tumor boundaries, local textures, and global structures. Finally, the deep features are processed through pooling, dropout, and a fully connected layer to generate the classification results.

In FS-FCN, PWD modules are deployed across the multilevel encoding stages to improve feature preservation during downsampling. Two cascaded FSConv modules are placed after PWD4 to promote interactions between deep spatial features and different frequency components. Through their coordinated use, structural information and fine-grained textures in brain tumor MRI images are effectively modeled while the spatial resolution of the feature maps is progressively reduced.

### 3.2 Convolutional block architecture

**Figure 2:**
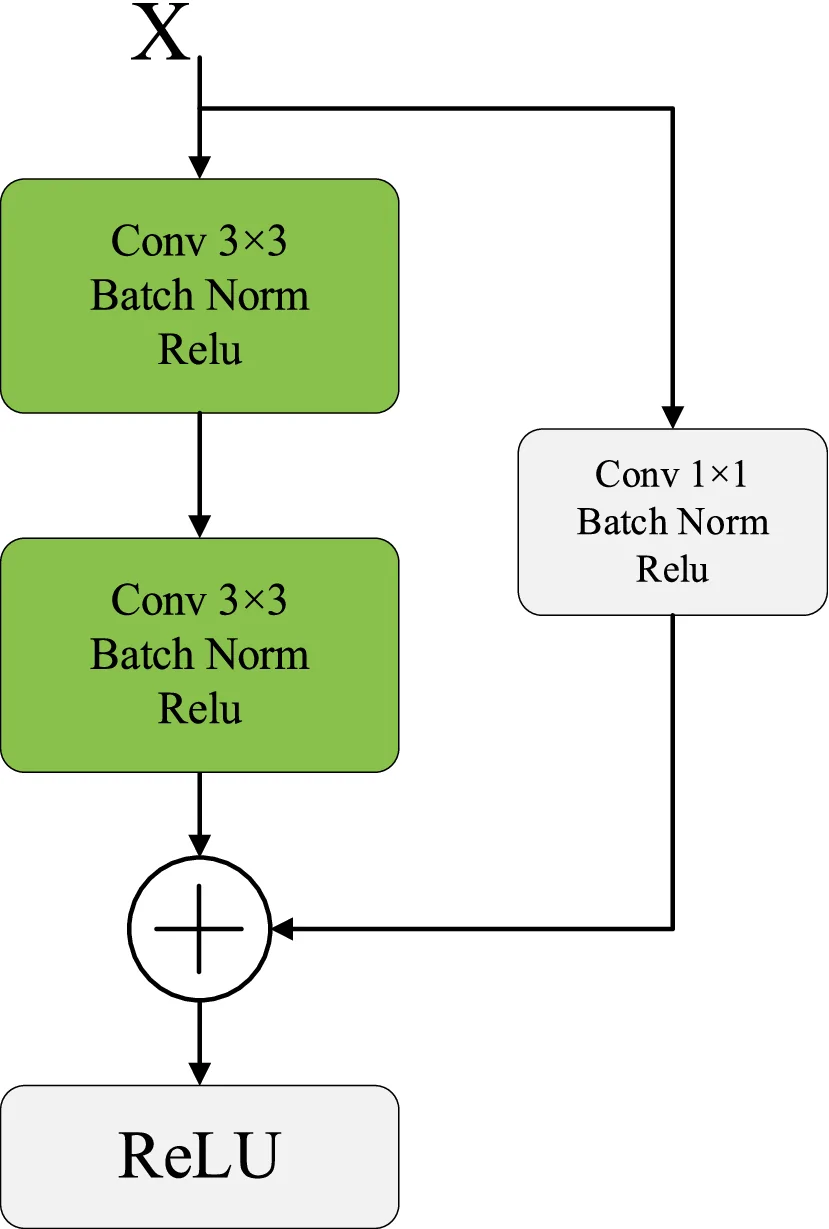
Structure of the convolutional block.

As shown in Figure 2, the convolutional block consists of a main branch and a residual mapping branch. In the main branch, the input feature X is sequentially processed by two 3 × 3 convolutional layers, each followed by BN and ReLU. Local spatial features, including intensity variations, edges, and textures, are thereby extracted. In the residual mapping branch, a 1 × 1 convolution followed by BN and ReLU is applied to align the number of input channels with that of the main-branch output. The outputs of the two branches are then added element-wise and passed through ReLU to produce the final output.

Through residual mapping, the input information is propagated directly to the output of the convolutional block. Local feature extraction is thereby enhanced, and this design helps alleviate gradient propagation difficulties associated with increasing network depth. After channel mapping, the residual features are effectively fused with the main-branch features, providing spatially informative input features for the subsequent PWD and FSConv modules.

### 3.3 PWD module

**Figure 3:**
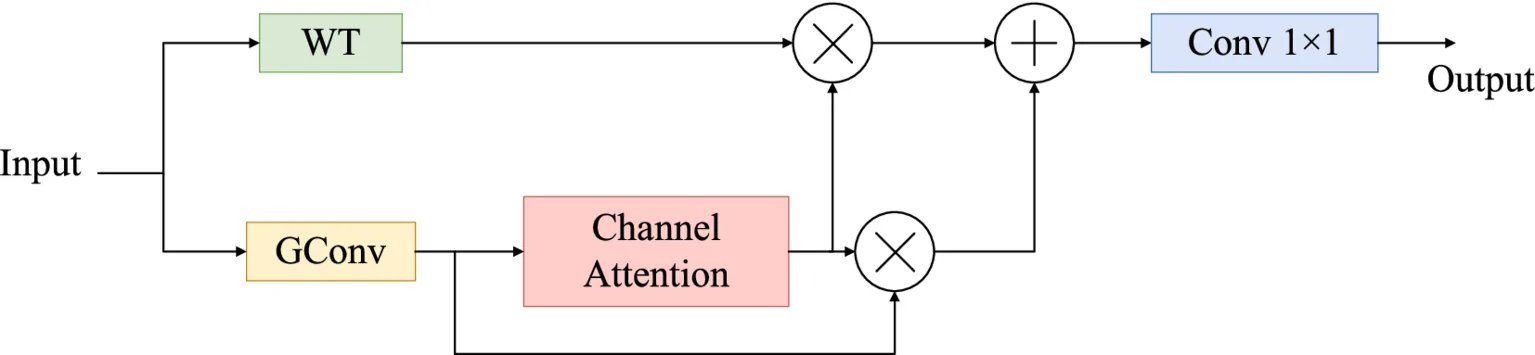
PWD module structure.

In deep CNNs, downsampling is used to reduce the spatial resolution of feature maps and enlarge the receptive field. However, conventional max-pooling and strided convolution mainly compress features in the spatial domain, potentially causing the loss of edge, texture, and local structural information. To preserve the fine-grained structural features required for brain tumor MRI classification, PWD modules are introduced into the encoding stages of FS-FCN. Wavelet- and spatial-domain features are modeled in parallel, allowing information to be better preserved during downsampling. As illustrated in Figure 3, the PWD module consists of parallel wavelet transform and grouped convolution branches. Channel attention is then used to adaptively modulate the features from both branches before feature fusion.

Given an input feature $X\in {\mathbb{R}}^{B\times C\times H\times W}$ , the Haar DWT is applied in the wavelet branch to decompose X into one low-frequency subband and three direction-specific high-frequency subbands, as shown in Equation 1:

$$\left\{X_{\mathrm{LL}},X_{\mathrm{LH}},X_{\mathrm{HL}},X_{\mathrm{HH}}\}={\mathrm{DWT}}_{\text{Haar}}(X\right)$$ (1)

Specifically, $X_{\mathrm{LL}}$ mainly preserves global structural information, whereas $X_{\mathrm{LH}}$ , $X_{\mathrm{HL}}$ and $X_{\mathrm{HH}}$ capture direction-specific edge and texture details. The four subbands are then concatenated along the channel dimension, as shown in Equation 2:

$$F_{w}=\text{Concat}(X_{\mathrm{LL}},X_{\mathrm{LH}},X_{\mathrm{HL}},X_{\mathrm{HH}})$$ (2)

This produces the wavelet-domain feature $F_{w}\in {\mathbb{R}}^{B\times 4C\times H/2\times W/2}$ . Meanwhile, in the spatial branch, the input feature is downsampled and local spatial information is extracted using a grouped convolution with a kernel size of 2 × 2 and a stride of 2, as shown in Equation 3:

$$F_{s}=\text{ReLU}(\mathrm{BN}({\text{GConv}}_{2\times 2,s=2,g=C}(X)))$$ (3)

Notably, $F_{s}$ and $F_{w}$ have the same spatial dimensions and number of channels, ensuring dimensional consistency for subsequent feature fusion.

Subsequently, channel weights are generated based on the results of global average pooling and global max pooling of the spatial branch features, as shown in Equation 4:

$$A=\sigma [f_{2}\;(\delta (f_{1}(\mathrm{GAP}(F_{s}\;))))+f_{2}\;(\delta (f_{1}\;(\mathrm{GMP}(F_{s}\;))))]$$ (4)

Here, $f_{1}$ and $f_{2}$ denote the 1 × 1 convolutional mappings shared by the average- and max-pooling branches, while δ and σ denote the ReLU and Sigmoid functions, respectively. The generated channel weights are then used to modulate both the spatial- and wavelet-domain features, as shown in Equations 5, 6:

$$F_{s}=A⊙F_{s}$$ (5)

$$F_{w}=\Gamma ⊙(A⊙F_{w}),$$ (6)

Here, ⊙ denotes element-wise multiplication, and $\Gamma \in {\mathbb{R}}^{1\times 4C\times 1\times 1}$ represents the scale adjustment coefficients applied to each channel of the wavelet branch. Finally, the features from the two branches are added element-wise, and channel fusion is performed using a 1 × 1 convolution, BN, and ReLU, as shown in Equation 7:

$$Y=\text{ReLU}[\mathrm{BN}({\text{Conv}}_{1\times 1}\;(F_{s}+F_{w}\;))]$$ (7)

The final output features are $Y\in {\mathbb{R}}^{B\times 2C\times H/2\times W/2}$ .

Through parallel downsampling and feature fusion, wavelet-domain frequency information and spatial responses from the grouped convolution branch are jointly utilized by PWD. Consequently, the feature-map resolution is reduced while global structures, local textures, and edge details are preserved for subsequent network layers.

### 3.4 FSConv module

Differences among brain tumor types in MRI images are often reflected in fine-grained features, including boundary morphology, local texture, and internal structure. Conventional convolutional operations mainly extract spatial-domain features and provide limited explicit modeling of different frequency components. Moreover, high-frequency responses associated with edges, textures, and local structures may be progressively weakened by multistage convolution and downsampling, thereby limiting the discrimination of visually similar tumor types. To address this limitation, FSConv modules are introduced into the deep feature extraction stage of FS-FCN. Spatial- and frequency-domain information is jointly extracted to improve the representation of local details and global structures.

**Figure 4:**
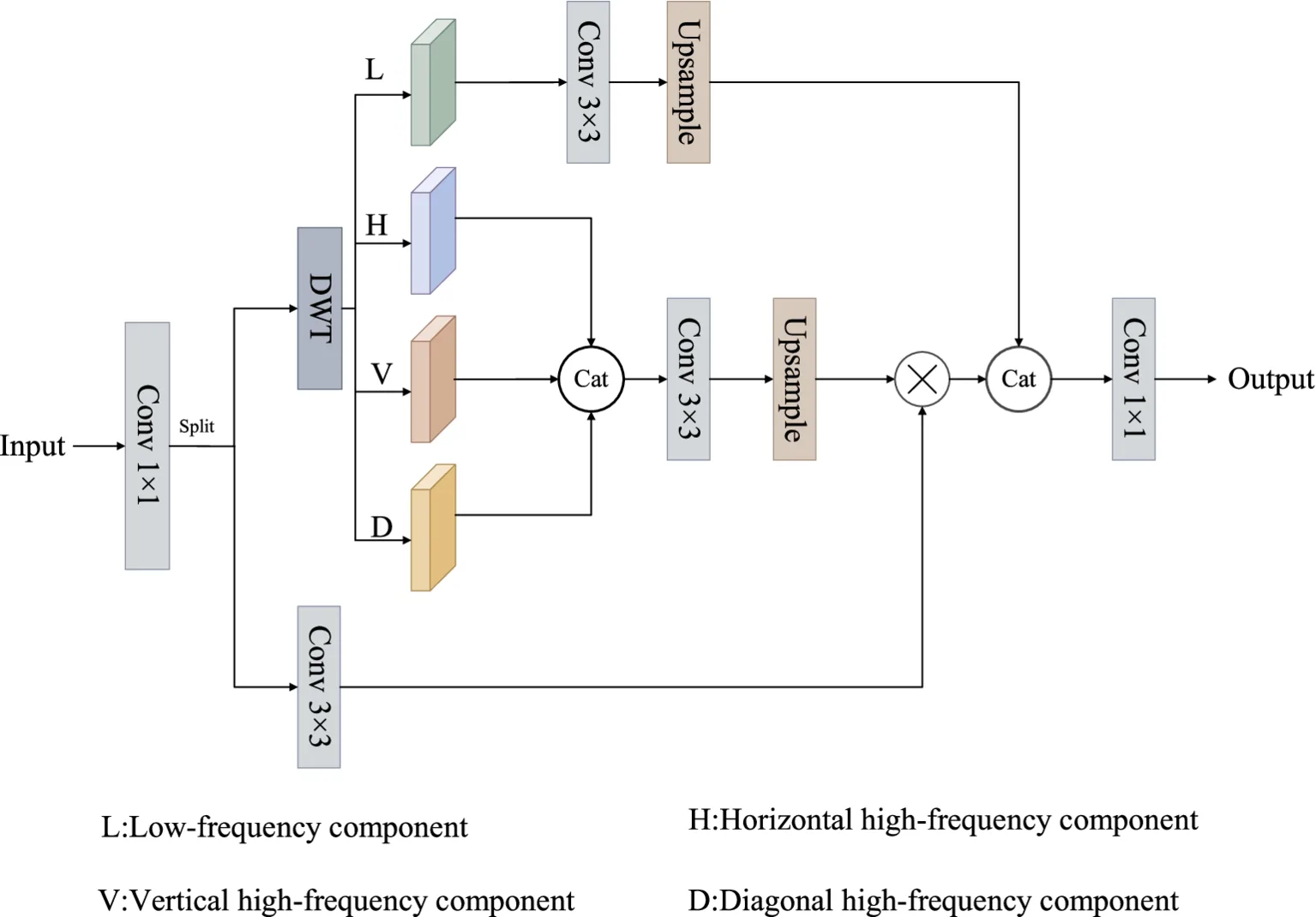
FSConv module structure.

As shown in Figure 4, FSConv mainly consists of channel expansion and splitting, spatial feature extraction, DWT, high-frequency feature extraction, low-frequency structural modeling, and feature fusion. After channel expansion, the input feature is divided into spatial- and frequency-domain branches. Spatial structural features are extracted by the spatial branch, whereas the frequency-domain branch employs the Haar DWT to obtain low-frequency structural information and high-frequency details. The resulting features are then processed and dimensionally aligned to enable spatial–frequency interaction and fusion, producing the final FSConv output.

For an input feature *X*∈ℝ^B × C × H × W^, the channels are first expanded via 1 × 1 convolutions to 2C channels. Subsequently, the expanded feature is divided into two parts along the channel dimension, which are used for spatial-domain feature extraction and frequency-domain information modeling, respectively.

For the frequency branch, the feature subset assigned to this branch after channel-wise splitting is decomposed by a discrete wavelet transform into one low-frequency component and three direction-specific high-frequency components, as shown in Equation 8:

$$\left\{L,H,V,D\}=\mathrm{DWT}(X_{f}\right)$$ (8)

Here, L represents the low-frequency component, while H, V, and D represent the horizontal, vertical, and diagonal high-frequency components, respectively. The three high-frequency components are concatenated along the channel dimension, as shown in Equation 9:

$$F_{h}=\text{Concat}\;\;(H,V,D)$$ (9)

The concatenated high-frequency features, which contain detailed responses from different directions, are further processed through 3 × 3 grouped convolutions, BN, and ReLU for feature extraction, as shown in Equation 10.

$$F_{h}^{\prime }=\text{ReLU}(\mathrm{BN}(\text{Conv}(F_{h}))$$ (10)

Next, $F_{h}^{\prime }$ is upsampled using a bilinear interpolation factor of 2 to restore it to the same spatial dimensions as the spatial branch, thereby providing size-consistent inputs for the subsequent element-wise interaction between high-frequency and spatial features.

The low-frequency component L primarily preserves the coarse structural and regional information of the input feature map. To extract structural features from the low-frequency component, it is first processed using a 3 × 3 convolution, BN, and ReLU, as shown in Equation 11:

$$F_{l}=\text{ReLU}(\mathrm{BN}(\text{Conv}(L))$$ (11)

Next, the low-frequency feature $F_{l}$ is upsampled using bilinear interpolation to restore it to the same spatial dimensions as the spatial branch. The upsampled low-frequency feature is denoted as $F_{l}^{\prime }$ and is used in the subsequent feature fusion stage to supplement the overall structural information.

At the same time, the feature subset assigned to the spatial branch after channel-wise splitting is processed by a 3 × 3 convolution, BN, and ReLU to extract local spatial structural features, as shown in Equation 12:

$$F_{s}=\text{ReLU}(\mathrm{BN}(\text{Conv}(X_{s}))$$ (12)

Subsequently, to leverage the guiding role of frequency-domain information on spatial features, this paper performs element-wise multiplicative fusion between the processed high-frequency features—after size restoration—and the spatial features, as shown in Equation 13:

$$F_{s}^{\prime }=F_{h}^{\prime }⊙F_{s}$$ (13)

Here, ⊙ denotes element-wise multiplication, which helps emphasize spatial responses associated with texture and edge information.

Finally, the modulated spatial features are concatenated with the upsampled low-frequency structural features along the channel dimension, as shown in Equation 14:

$$Y=\text{Concat}({\hat{F}}_{s},{\hat{F}}_{l})$$ (14)

Subsequently, the concatenated features are fused through a 1 × 1 convolution to produce the FSConv output. Through the aforementioned frequency–spatial joint modeling process, FSConv is able to fully leverage high-frequency detail information, low-frequency structural information, and spatial features, thereby enhancing the network’s ability to capture fine-grained differences in brain tumor MRI images.

## 4 Experiments and results

In this section, FS-FCN is experimentally evaluated for brain tumor MRI classification. The dataset, training settings, and evaluation metrics are first described, followed by the main results on the three-class classification task. In addition, an extended four-class experiment was conducted to further assess the applicability of the model across different classification settings.

### 4.1 Dataset and experimental setup

A publicly available T1-weighted contrast-enhanced MRI (CE-MRI) dataset was used for the main experiments. The dataset contains 3,064 brain tumor MRI images, including 1,426 glioma images, 708 meningioma images, and 930 pituitary tumor images. To improve the reliability of the experimental results, stratified five-fold cross-validation was used for model training and evaluation. The dataset was partitioned into five folds while approximately preserving the class distribution in each fold. The random seed was set to 41 to ensure reproducibility. In each iteration, four folds were used for training, and the remaining fold was used for validation. This process was repeated five times so that each fold served as the validation set once. The final classification performance was reported as the mean and standard deviation of the five validation results. In addition, supplementary experiments were performed on a four-class dataset containing 3,264 images, including 926 glioma images, 937 meningioma images, 901 pituitary tumor images, and 500 no-tumor images. Five-fold cross-validation was not used in these supplementary experiments.

All input images were resized to 224 × 224 pixels. They were then converted into tensors, with pixel values scaled to [0,1]. All three input channels were normalized using a mean of 0.1567 and a standard deviation of 0.1686. To increase training-sample diversity and reduce overfitting, random rotation and horizontal flipping were applied during training. The rotation angle was randomly selected from $\left[-15^{\circ },15^{\circ }\right]$ , and horizontal flipping was performed with a probability of 0.5. These augmentation operations were applied only to the training set, whereas no random transformations were applied to the validation set.

All experiments were conducted on Windows Server 2022 Standard using an Intel Xeon Gold 6436Y processor and an NVIDIA GeForce RTX 4090D GPU. The model was trained end-to-end using PyTorch. The number of base channels was set to 32, and the batch size was set to 16. The dropout rates before the classifier and after the two cascaded FSConv modules were set to 0.3958 and 0.0167, respectively. The Adam optimizer and cross-entropy loss were used for model training. The initial learning rate was set to $5.5435\times 10^{-4}$ , and the maximum number of training epochs was set to 100. In addition, ReduceLROnPlateau and early stopping were employed to adjust the learning rate and terminate training when the validation performance no longer improved.

### 4.2 Evaluation metrics

To comprehensively evaluate the classification performance of FS-FCN, four quantitative metrics were used: Accuracy, Precision, Recall, and F1-score. Accuracy measures the proportion of correctly classified samples among all samples. Precision represents the proportion of samples predicted as a given class that actually belong to that class, whereas Recall represents the proportion of samples from that class that are correctly identified. F1-score is defined as the harmonic mean of Precision and Recall and provides a balanced assessment of classification performance (27). These metrics are calculated using Equations 15–18:

$$\text{Accuracy}=\frac{\mathrm{TP}+\mathrm{TN}}{\mathrm{TP}+\mathrm{TN}+\mathrm{FP}+\mathrm{FN}}$$ (15)

$$\text{Precision}=\frac{\mathrm{TP}}{\mathrm{TP}+\mathrm{FP}}$$ (16)

$$\text{Recall}=\frac{\mathrm{TP}}{\mathrm{TP}+\mathrm{FN}}$$ (17)

$$F1-\text{score}=\frac{2\times \text{Precision}\times \text{Recall}}{\text{Precision}+\text{Recall}}$$ (18)

Here, TP , TN , FP , and FN denote the numbers of true positives, true negatives, false positives, and false negatives, respectively. Specifically, they represent positive samples correctly classified as positive, negative samples correctly classified as negative, negative samples incorrectly classified as positive, and positive samples incorrectly classified as negative.

In addition to the quantitative metrics described above, confusion matrices, receiver operating characteristic (ROC) curves, and the corresponding area under the curve (AUC) values were used to further evaluate the classification results (28, 29). A confusion matrix presents the correspondence between the true and predicted labels, thereby showing the numbers of correctly classified and misclassified samples for each class. The ROC curve plots the false positive rate on the x-axis and the true positive rate on the y-axis, while the AUC measures the model’s discriminative ability across different classification thresholds. The AUC was calculated separately for each class, and the macro-averaged AUC was used to assess the model’s overall discriminative performance.

To assess the performance differences between FS-FCN and the comparison models, paired comparisons of fold-wise accuracy were performed using the same five-fold data splits. The 95% confidence interval (CI) for the mean paired difference was then calculated based on Student’s t-distribution, as shown in Equation 19:

$$95\%\mathrm{CI}=\bar{d}\pm t_{0.975,n-1}\frac{s_{d}}{\sqrt{n}}$$ (19)

Here, n = 5, and $\bar{d}$ and $s_{d}$ denote the mean and sample standard deviation of the fold-wise accuracy differences, respectively. $t_{0.975,n-1}$ denotes the critical value of Student’s t-distribution with (n-1) degrees of freedom. In Table 1, ΔAcc represents the mean improvement in the accuracy of FS-FCN over each comparison model, expressed in percentage points. The difference in accuracy was considered statistically significant if the corresponding 95% confidence interval did not include zero.

**Table 1:** Performance comparison of different models.

Model	Precision (%)	Recall (%)	F1-score (%)	Accuracy (%)	ΔAcc (95% CI)
ResNet50 (31)	96.83 ± 0.97	96.94 ± 0.91	96.87 ± 0.91	97.23 ± 0.79	1.30 [0.61, 2.00]
ViT-B/16 (32)	95.67 ± 0.72	95.25 ± 0.56	95.44 ± 0.58	95.92 ± 0.60	2.61 [2.11, 3.10]
ConvNeXt-Tiny (33)	95.36 ± 0.56	95.58 ± 1.14	95.43 ± 0.79	95.95 ± 0.58	2.58 [2.11, 3.04]
Swin-T (34)	96.67 ± 0.90	96.39 ± 0.55	96.50 ± 0.60	96.93 ± 0.57	1.60 [0.88, 2.31]
EfficientNetV2-S (35)	96.66 ± 0.96	96.68 ± 0.72	96.67 ± 0.82	97.03 ± 0.72	1.50 [0.47, 2.52]
MedViT-Small (36)	95.63 ± 0.80	95.50 ± 0.66	95.54 ± 0.71	96.08 ± 0.64	2.45 [1.90, 3.00]
Proposed	98.42 ± 0.34	98.26 ± 0.51	98.33 ± 0.40	98.53 ± 0.35	—

### 4.3 Experimental results

To evaluate the effectiveness of the proposed FS-FCN model for brain tumor MRI classification, its performance was first assessed using stratified five-fold cross-validation on a three-class brain tumor dataset. The dataset included glioma, meningioma, and pituitary tumor images. Precision, recall, F1-score, and accuracy were used to comprehensively evaluate the model’s classification performance across different classes. The results were reported as the mean ± standard deviation obtained from five-fold cross-validation. The results are presented in Table 2.

**Table 2:** Classification performance of FS-FCN on the three-class task.

Tumor class	Precision (%)	Recall (%)	F1-score (%)	Accuracy (%)
Glioma	98.75 ± 0.79	99.02 ± 0.29	98.88 ± 0.31	98.53 ± 0.35
Meningioma	97.58 ± 1.03	96.19 ± 1.77	96.87 ± 0.79	
Pituitary	98.93 ± 0.53	99.57 ± 0.45	99.25 ± 0.29	

As shown in Table 2, strong performance was achieved by FS-FCN in the three-class classification task, with a mean five-fold accuracy of 98.53% ± 0.35%. The highest class-specific performance was obtained for pituitary tumors, with precision, recall, and F1-score values of 98.93% ± 0.53, 99.57% ± 0.45, and 99.25% ± 0.29%, respectively. For gliomas, precision, recall, and F1-score values of 98.75% ± 0.79, 99.02% ± 0.29, and 98.88% ± 0.31% were achieved, respectively. For meningiomas, the corresponding values were 97.58% ± 1.03, 96.19% ± 1.77, and 96.87% ± 0.79%, respectively. Although these values were slightly lower than those for the other two classes, satisfactory classification performance was still achieved. Overall, high performance was observed across all three brain tumor classes. The generally small standard deviations across the five folds further support the effectiveness and consistency of the proposed model for three-class brain tumor MRI classification.

**Figure 5:**
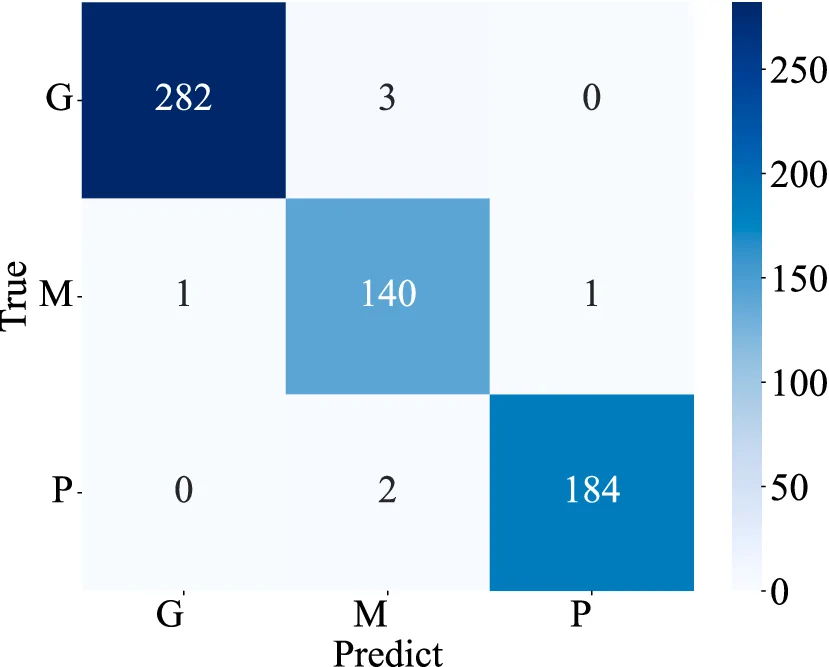
Confusion matrix of FS-FCN on the validation set.

Figure 5 presents the confusion matrix for the fold with the highest validation accuracy in the stratified five-fold cross-validation. G, M, and P denote glioma, meningioma, and pituitary tumors, respectively. Most predictions were concentrated along the main diagonal, indicating strong overall classification performance. Of the 285 glioma samples, only 3 were misclassified as meningiomas. Of the 142 meningioma samples, 1 was misclassified as a glioma and 1 as a pituitary tumor. Of the 186 pituitary tumor samples, only 2 were misclassified as meningiomas. The small number of misclassified samples indicates that the model can effectively distinguish among the three tumor classes.

**Figure 6:**
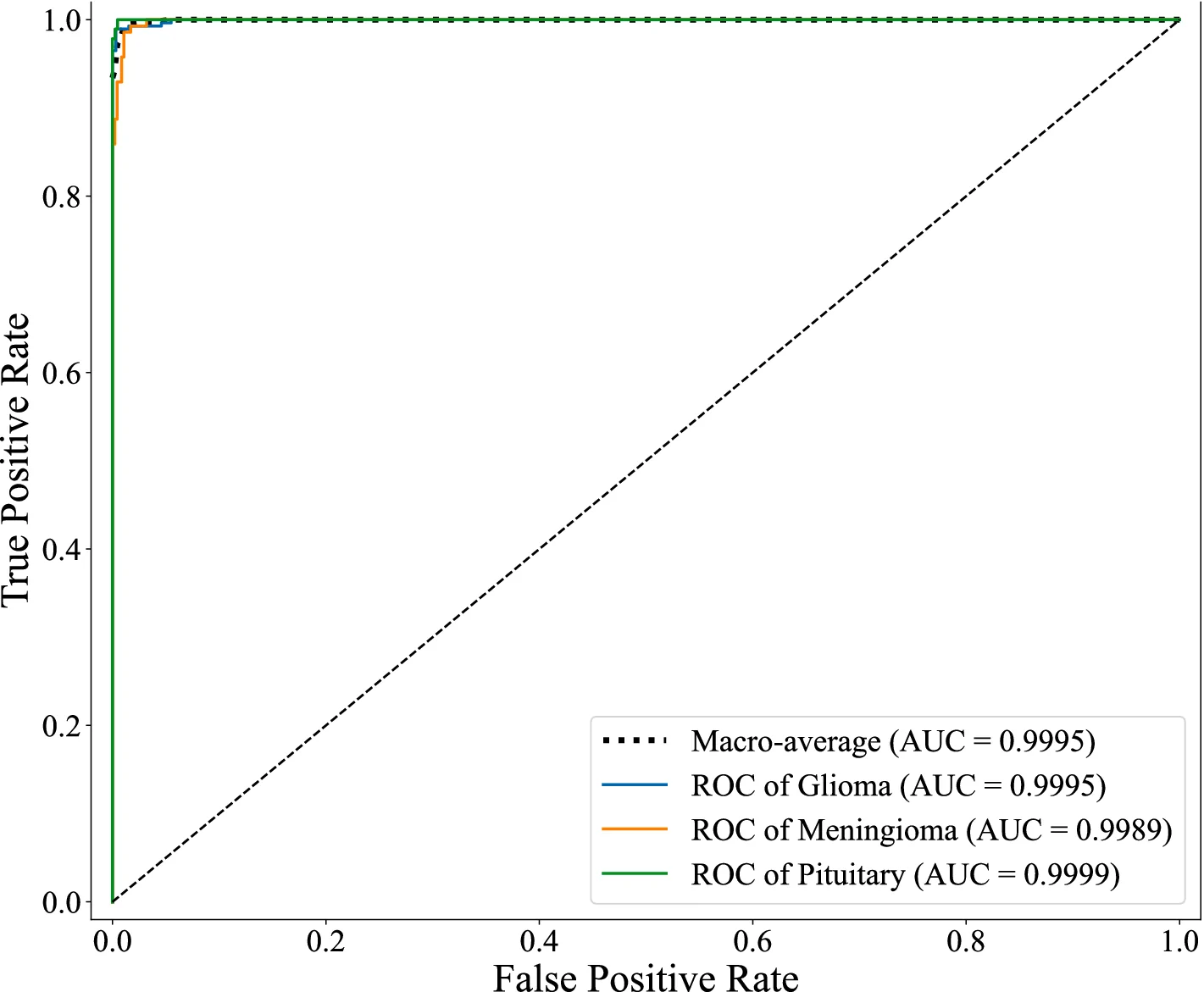
ROC curves and corresponding AUC values of FS-FCN for each class.

To further evaluate the classification performance and discriminative ability of FS-FCN across different classes, ROC curves and the corresponding AUC values were generated using the fold with the highest validation accuracy in the stratified five-fold cross-validation. The results are presented in Figure 6. The ROC curves for all three tumor classes were close to the upper-left corner and far from the diagonal line representing random classification, indicating strong discriminative ability across different classification thresholds. Specifically, the AUC values for gliomas, meningiomas, and pituitary tumors were 0.9995, 0.9989, and 0.9999, respectively, and the macro-average AUC was 0.9995. These results demonstrate that FS-FCN can effectively distinguish among the three types of brain tumors.

### 4.4 Supplementary four-class classification experiment

To further validate the applicability of FS-FCN across different classification settings, a supplementary experiment was conducted on a four-class dataset consisting of glioma, meningioma, pituitary tumor, and no-tumor images. The results are presented in Table 3. An overall accuracy of 97.10% was achieved, indicating that discriminative features could be effectively learned across the four classes. The best performance was obtained for pituitary tumors, with precision, recall, and F1-score all reaching 100%. For the no-tumor class, a precision of 96.15% and a recall of 100% were achieved, indicating that all no-tumor samples were correctly identified. Despite the visual similarity between gliomas and meningiomas, F1-scores of 95.29 and 95.56% were obtained, respectively. These results demonstrate that FS-FCN can effectively distinguish brain tumor types and is applicable to four-class classification with no-tumor samples.

**Table 3:** Classification performance of FS-FCN on the four-class classification task.

Tumor class	Precision (%)	Recall (%)	F1-score (%)	Accuracy (%)
Glioma	98.29	92.47	95.29	97.10
Meningioma	93.85	97.34	95.56	
No tumor	96.15	100	98.04	
Pituitary	100	100	100	

## 5 Discussion

In this section, the classification performance of FS-FCN is evaluated through ablation studies, an analysis of FSConv placement, visualization analyses, and comparisons with other models.

### 5.1 Ablation experiment

In this section, ablation studies were conducted to evaluate the effects of different PWD and FSConv configurations on classification performance. The results are presented in Table 4, with all metrics reported as the mean ± standard deviation from five-fold cross-validation. The PWD-only and FSConv-only configurations achieved mean accuracies of 95.95% ± 0.99 and 97.26% ± 0.70%, respectively, whereas the complete FS-FCN achieved 98.53% ± 0.35%. Compared with the complete model, the removal of FSConv reduced accuracy by 2.58 percentage points, whereas the removal of PWD reduced accuracy by 1.27 percentage points. These results indicate that FSConv made a greater contribution to classification performance under the current experimental setting, while PWD provided complementary features. When both modules were combined, the highest precision, recall, F1-score, and accuracy were achieved, together with smaller standard deviations. This finding suggests that the combined use of PWD and FSConv improves the overall classification performance and stability of the model.

**Table 4:** Ablation results for different module configurations.

Module configuration	Precision (%)	Recall (%)	F1-score (%)	Accuracy (%)
PWD	95.77 ± 1.40	95.37 ± 0.93	95.51 ± 1.08	95.95 ± 0.99
FSConv	97.14 ± 0.86	96.77 ± 0.67	96.93 ± 0.73	97.26 ± 0.70
FS-FCN	98.42 ± 0.34	98.26 ± 0.51	98.33 ± 0.40	98.53 ± 0.35

### 5.2 Validation of FSConv placement

The effect of FSConv placement at different network levels was further investigated. Two cascaded FSConv modules were inserted after either PWD1 or PWD4, forming shallow and deep placement schemes, respectively. The deep placement corresponds to the FS-FCN architecture shown in Figure 1. The same data split, image preprocessing, training strategy, and hyperparameter settings were used for both schemes. For the shallow placement, the channel dimensions at the FSConv output were adjusted to connect it to the subsequent encoding stage. Except for the FSConv placement and the required dimensional adjustments, the network architecture and experimental settings remained unchanged.

As shown in Table 5, when two cascaded FSConv modules were placed after PWD1, an accuracy of 97.71% was achieved, with precision, recall, and F1-score values of 97.26, 97.82, and 97.52%, respectively. When the modules were placed after PWD4, the accuracy increased to 98.53%, while precision, recall, and F1-score reached 98.42, 98.26, and 98.33%, respectively. All metrics were higher than those obtained with the shallow placement. This improvement may be attributed to the richer structural and class-discriminative information contained in deep features, which facilitates the fusion of frequency- and spatial-domain features by FSConv. Therefore, two cascaded FSConv modules were placed after PWD4 in the proposed model. These results support the selection of the deep placement for FSConv.

**Table 5:** Classification results for different FSConv placements.

FSConv placement	Precision (%)	Recall (%)	F1-score (%)	Accuracy (%)
Shallow placement	97.26	97.82	97.52	97.71
Deep placement	98.42	98.26	98.33	98.53

### 5.3 Visualization analysis

**Figure 7:**
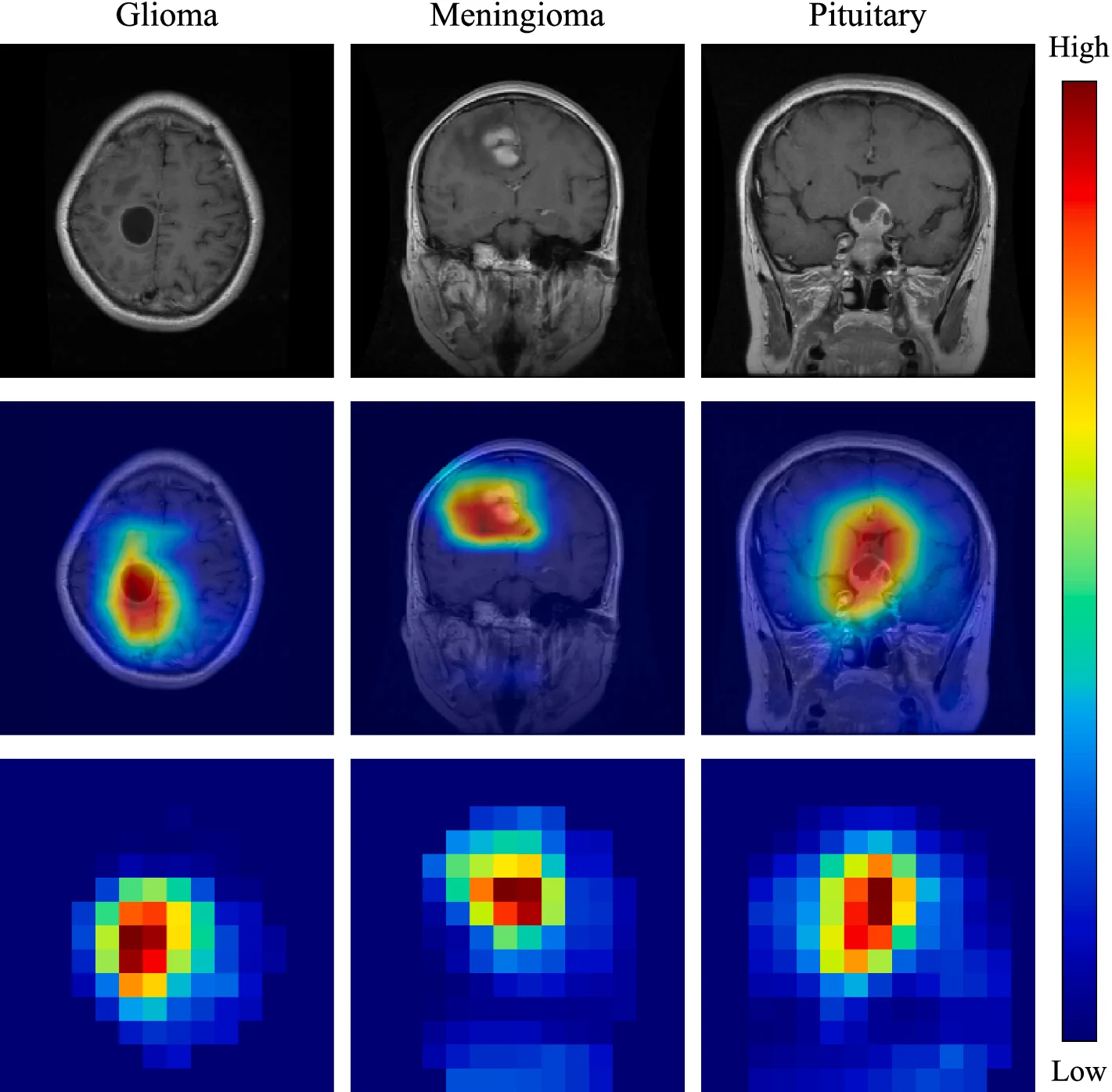
Visualization results of FS-FCN.

Gradient-weighted class activation mapping (Grad-CAM) (30) and deep feature response maps (DFRMs) were further employed to qualitatively analyze the class-discriminative regions and deep feature responses of FS-FCN during brain tumor MRI classification. The results are presented in Figure 7. The first row shows the original MRI images, the second row shows the Grad-CAM heatmaps, and the third row shows the DFRM. Specifically, Grad-CAM reflects the primary regions attended to by the model when making classification decisions. The DFRM is generated by extracting the output features of the second FSConv module and averaging them along the channel dimension, thereby showing the overall distribution of deep feature responses at different spatial locations. In the three examples of glioma, meningioma, and pituitary tumor, the high-response regions in the Grad-CAM heatmaps are mainly distributed over the lesions and adjacent areas. The strong responses in the DFRM also show a certain degree of spatial correspondence with some abnormal structures. From the perspectives of class-discriminative regions and deep feature distributions, these results provide qualitative evidence that the model uses tumor-related structural and textural information for classification.

### 5.4 Comparison with representative deep learning models

#### 5.4.1 Quantitative comparison

To further evaluate the classification performance of FS-FCN, comparisons were conducted with ResNet50, ViT-B/16, ConvNeXt-Tiny, Swin-T, EfficientNetV2-S, and MedViT-Small. The results are presented in Table 1. The same data splits, image preprocessing methods, and evaluation procedure were used for all models, while model-specific pretraining methods, parameter-freezing strategies, and training configurations were adopted according to their respective architectures. All performance metrics are reported as the mean ± standard deviation obtained from five-fold cross-validation. The ΔAcc values and their 95% confidence intervals were calculated using the paired analysis method described in Section 4.2.

FS-FCN achieved precision, recall, F1-score, and accuracy values of 98.42% ± 0.34, 98.26% ± 0.51, 98.33% ± 0.40, and 98.53% ± 0.35%, respectively, outperforming all six comparison models. Compared with the comparison models, FS-FCN achieved a mean accuracy improvement ranging from 1.30 to 2.61 percentage points. The 95% confidence intervals for all pairwise differences excluded zero, indicating that the improvements were statistically significant. ResNet50 was the best-performing comparison model, achieving an accuracy of 97.23% ± 0.79%. Compared with ResNet50, FS-FCN improved accuracy by 1.30 percentage points, with a 95% confidence interval of [0.61, 2.00]. The higher mean accuracy and smaller standard deviation suggest better classification performance and greater stability across the five folds, respectively.

#### 5.4.2 Grad-CAM comparison

**Figure 8:**
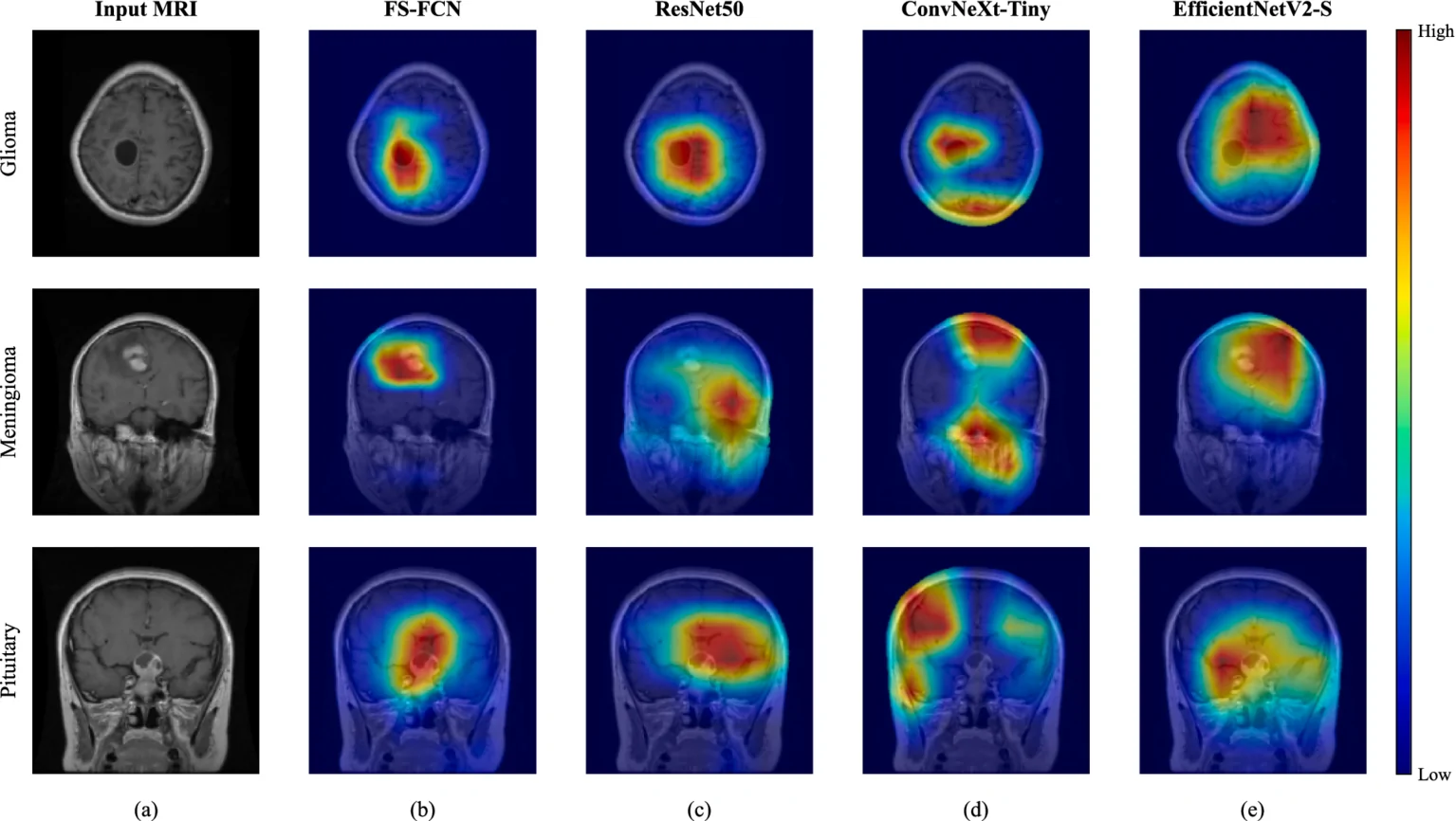
Grad-CAM heatmaps of different models for three brain tumor classes. **(a)** Original image. **(b)** FS-FCN. **(c)** ResNet50. **(d)** ConvNeXt-Tiny. **(e)** EfficientNetV2-S.

To visually compare the feature regions emphasized by different models, Grad-CAM visualizations were generated for FS-FCN, ResNet50, ConvNeXt-Tiny, and EfficientNetV2-S. The results are presented in Figure 8. Red regions indicate greater model attention, whereas blue regions indicate lower attention. For FS-FCN, the response regions were more concentrated in the glioma, meningioma, and pituitary tumor images. Its high-response regions showed clear spatial correspondence with the tumor lesions and adjacent areas. In contrast, the response regions of the other models were relatively dispersed, with some attention directed toward surrounding or non-lesion regions. These qualitative results suggest that FS-FCN tends to produce more spatially concentrated responses in tumor-related regions, which may support the extraction of discriminative features.

### 5.5 Comparison with other methods

In this section, FS-FCN was compared with representative methods recently evaluated on the Cheng brain tumor MRI dataset for three-class classification. The results are presented in Table 6. FS-FCN achieved precision, recall, F1-score, and accuracy values of 98.42, 98.26, 98.33, and 98.53%, respectively. These values were higher than the corresponding results reported for the other methods. Compared with the method proposed by Rahman et al., which achieved relatively high accuracy, FS-FCN improved accuracy by 0.40 percentage points. The observed performance improvement may be associated with the complementary roles of PWD and FSConv, as supported by the ablation results. During downsampling, PWD preserves structural, edge, and texture information at different frequencies, thereby reducing the loss of important details. FSConv further integrates frequency- and spatial-domain information in deep features, enhancing the differences among tumor classes in terms of boundaries, textures, and overall morphology. Therefore, more discriminative brain tumor features can be extracted by FS-FCN, resulting in better overall classification performance.

**Table 6:** Comparison of classification results between FS-FCN and other methods.

Reference	Precision (%)	Recall (%)	F1-score (%)	Accuracy (%)
Shaik et al. (37)	96.14	95.99	96.03	96.51
Tummala et al. (38)	—	—	—	97.71
Saurav et al. (39)	97.07	96.95	97.00	97.23
Khan et al. (40)	95.00	94.00	94.00	95.10
Montoya et al. (41)	—	—	—	97.00
Rahman et al. (42)	97.74	98.05	97.80	98.13
Alhassan et al. (43)	97.98	97.20	97.58	97.59
Li et al. (44)	97.80	98.20	98.00	98.04
Proposed	98.42	98.26	98.33	98.53

## 6 Conclusion

To address the insufficient use of frequency information by conventional convolutional neural networks and the loss of details during downsampling, FS-FCN was developed for brain tumor MRI classification. Through joint frequency- and spatial-domain modeling, structural and textural details are preserved during feature compression, enabling the joint representation of local textures and global structures. On the Cheng three-class brain tumor MRI dataset, FS-FCN achieved a mean accuracy of 98.53% ± 0.35% through five-fold cross-validation. Ablation experiments confirmed the effectiveness of combining PWD and FSConv, while the placement experiment showed that the best overall performance was achieved when two cascaded FSConv modules were placed after PWD4. Quantitative comparisons demonstrated superior overall classification performance, and qualitative Grad-CAM visualizations showed more concentrated responses in tumor-related regions and adjacent areas. The four-class experiment further supported the applicability of FS-FCN under different class settings. Overall, these results indicate that joint frequency- and spatial-domain modeling provides an effective feature-learning approach for brain tumor MRI classification.

Clinically, FS-FCN could serve as an auxiliary image interpretation tool for the preliminary classification of glioma, meningioma, and pituitary tumors, while Grad-CAM could visualize the regions emphasized by the model. However, FS-FCN cannot currently replace the comprehensive diagnosis provided by radiologists. Although the model was evaluated on two public two-dimensional MRI datasets, the Cheng dataset used in the main experiments contains only contrast-enhanced T1-weighted images. Its applicability to other MRI sequences, such as T2-weighted and FLAIR images, therefore requires further validation. Moreover, the available data sources and imaging conditions were limited, and independent multicenter external validation was not performed. Consequently, the generalizability of the model across different scanners, imaging parameters, and patient populations remains uncertain. The Grad-CAM and DFRM analyses were also primarily qualitative, without quantitative evaluation based on lesion annotations. Future work will therefore include multicenter validation using clinical data from multiple MRI sequences and imaging conditions, together with more systematic quantitative interpretability analyses, to further evaluate the robustness, generalizability, clinical reliability, and feasibility of FS-FCN.

## Data Availability

The original contributions presented in the study are included in the article/supplementary material, further inquiries can be directed to the corresponding author.
